# Supplementary material for: Impact of seed node position on network robustness under localized attacks
Source: PLoS One. 2026 Sep 22;21(9):e0358769. doi: 10.1371/journal.pone.0358769 (PMC13596812; doi:10.1371/journal.pone.0358769)
Supplement: Supplementary file 1 [file pone.0358769.s002.pdf]

Response to Reviewer

We would like to thank the Editor and the Reviewers for their careful reading of our manuscript and for their constructive and helpful comments. We have revised the manuscript accordingly and provided detailed responses to each comment below.

Reviewer #1:

1) Include a clearer comparison with related work. Situate LAVI more explicitly within the existing literature on network vulnerability and robustness, explaining what gaps it fills compared to previously proposed indices.

Ans. We revised the Introduction to include a clearer comparison with related work and to explicitly position LAVI within the existing literature on network robustness and vulnerability by clarifying the gap addressed by the proposed index. The corresponding changes were added in lines 29–65 on page 2.

2) Expand the methodological details. The description of the LAVI metric and the localized attack model is concise. Adding step-by-step explanations or a small numerical example would significantly improve clarity and reproducibility. To improve clarity and reproducibility, I strongly recommend adding pseudocode for the main procedures.

Ans. We added Table 1 to provide pseudocode and a step-by-step description of the main procedures, improving the clarity and reproducibility of the proposed method.

3) Clarify the motivation for the two removal strategies. The rationale behind the "random-case" and "worst-case" removal orders could be better justified, including a clearer explanation of the real-world scenarios each scheme is intended to represent.

Ans. We added a brief explanation clarifying the motivation of the random-case and worst-case removal strategies by relating them to stochastic damage scenarios and adversarial localized attacks, respectively (lines 123–128 on page 4).

4) Strengthen the discussion of centrality metrics. The manuscript could benefit from a more thorough explanation of why classical centrality measures fail to correlate with robustness under localized attacks, possibly with illustrative examples or theoretical arguments.

Ans. We expanded the discussion to explain that high-degree or high-betweenness nodes perform poorly under localized attacks, while high-closeness nodes show relatively better performance in some cases. We also discussed the behavior of LAVI in these examples and emphasized the fundamental difference between classical targeted attacks and localized attack propagation mechanisms. Specifically, the background motivation for degree- and betweenness-based targeted attacks is added in lines 29–34 on page 2, while the detailed discussion under localized attack scenarios is provided in lines 236–258 on page 8.

5) Discuss exceptional cases more deeply. For networks in which LAVI shows weak correlations (e.g., the bio-diseasome network), expand the analysis to explore structural properties. I also would be curious about the results on a criminal network. Please see https://www.doi.org/10.1109/TIFS.2023.3256706.

Ans. We expanded the analysis of the bio-diseasome network by adding network visualization and structural discussion to better explain the weak correlation observed for LAVI (lines 225–258 on pages 7–8). In addition, we discussed the potential application of LAVI to criminal networks as a future research direction in the Discussion section (lines 306–315 on pages 9–10).

6) Broaden the discussion section. The discussion is relatively short. Expanding it to cover practical implications, potential applications, and concrete examples of how LAVI could support infrastructure planning or risk assessment would strengthen the impact of the study. Explicitly addressing limitations would improve transparency and balance.

Ans. We expanded the Discussion section by adding practical implications and application examples of LAVI, as well as an explicit discussion of methodological limitations and computational considerations, in order to improve the balance and transparency of the manuscript (lines 288–315 on pages 9–10).

Reviewer #2:

1) The manuscript should include a more explicit justification for the choice of the link removal count as the core of LAVI, linking it more directly to practical implications for network functionality and resilience (e.g., service disruption, flow capacity) beyond just structural connectivity.

Ans. We added an explicit justification of the link removal count in the Method section by explaining its interpretation as a measure of cumulative connectivity loss during localized attack propagation (lines 134–143 on page 4). In addition, we clarified in the Discussion that LAVI is formulated from a structural connectivity perspective and discussed its relation to practical network functionality and potential extensions toward flow- and capacity-based models (lines 288–295 on page 9).

2) The interpretation of results for the bio-diseasome network is somewhat ambiguous, as LAVI performs weakly there. A deeper discussion on the potential reasons (e.g., community structure, degree distribution) and the implications for LAVI's generalizability across diverse network types would be valuable.

Ans. We expanded the discussion of the bio-diseasome network by adding a detailed structural analysis and visualization to explain the weak correlation observed for LAVI. We also discussed the implications of modular and bridge-dominated network structures for the generalizability of the proposed metric across different network types (lines 225–258 on pages 7–8).

3) While the computational complexity is briefly noted, a discussion on the practical scalability and potential challenges of applying LAVI to extremely large-scale real-time systems (e.g., global internet) would strengthen the practical utility section.

Ans. We expanded the Discussion section by adding a scalability-oriented discussion on the practical challenges of applying LAVI to large-scale and real-time network systems, including computational cost, dynamic topology changes, and data availability issues (lines 296–305 on page 9).

4) The manuscript could be improved by adding a more detailed comparison or discussion on how LAVI specifically differs from or complements existing notions of "node influence" in failure propagation models beyond just centrality measures.
<u>Ans.</u> We expanded the Introduction to clarify how LAVI differs from and complements existing node influence and failure propagation frameworks by emphasizing the fundamental distinction between global diffusion-based influence models and localized attack propagation scenarios (lines 29–65 on page 2).

5) The invulnerability of networks is a hotspot in recent study, and some correlative discussions on the robustness of seeds may further improve the significance of this work, e.g., Swarm and Evolutionary Computation, vol. 87, p. 101542, 2024 and Knowledge-Based Systems, vol. 275, p. 110677, 2023.
<u>Ans.</u> We added recent studies on robust and influential seed identification to the Introduction and discussed their relation to the present work in the context of network robustness and vulnerability analysis (lines 29–44 on page 2).
