# Supplementary material for: Impact of seed node position on network robustness under localized attacks
Source: PLoS One. 2026 Sep 22;21(9):e0358769. doi: 10.1371/journal.pone.0358769 (PMC13596812; doi:10.1371/journal.pone.0358769)
Supplement: Supplementary file 2 [file pone.0358769.s003.pdf]

Response to Reviewers

We sincerely thank the editor and the reviewers for their careful evaluation of our manuscript and for their constructive comments. We have revised the manuscript accordingly and summarize the main changes below, followed by point-by-point responses.

In this revision, we have made the following major changes:

1. We improved the clarity of the manuscript by revising the Introduction and Discussion to better explain the novelty of the proposed method and the role of network topology.
2. We expanded the Discussion section to explicitly address how network topology influences the performance of LAVI, including its behavior in heterogeneous and modular networks.
3. We revised the presentation of the algorithm by converting Table 1 into a pseudocode-style figure (Figure 1), as the journal template does not support a dedicated algorithm environment, thereby improving readability and clarity of the computational procedure.
4. We made minor edits throughout the manuscript to improve clarity, consistency, and readability.

We hope that these revisions satisfactorily address all concerns.

Response to the Editor

Comments:

Major revision is needed in the following aspects: Further justification and discussion of how network topology may influence the behavior of the index. Clearer description of innovation compared to the references.

Response:

We thank the editor for highlighting these important points.

Comment 1: Further justification and discussion of how network topology may influence the behavior of the index.

Response: **We added discussions in Lines 283–300 on Page 9. To clarify the role of network topology, we expanded the Discussion section to explicitly explain how the performance of LAVI depends on structural properties of the network. In particular, we added new paragraphs that discuss why LAVI performs well in heterogeneous networks such as scale-free networks, where early link removal strongly affects global connectivity, and why its predictive power is reduced in networks with strong modular or bridge-dependent structures, where fragmentation is governed by specific inter-community connectors. These additions make explicit that the effectiveness of LAVI is inherently topology dependent.**

Comment 2: *Clearer description of innovation compared to the references.*

Response: **We revised the Introduction (Lines 67–75, Pages 2–3) to more clearly describe the novelty of this study. We clarified that, unlike conventional centrality measures that rely on static structural importance, the proposed LAVI incorporates propagation dynamics originating from a single seed node and enables seed-level vulnerability assessment under localized attack dynamics. We also revised the surrounding text to better distinguish our approach from existing centrality-based and optimization-based methods. This revision clarifies the distinction from prior work and addresses the editor’s concern regarding the novelty of the study.**

Response to the Reviewers

Reviewer #1

We thank the reviewer for the positive evaluation and helpful suggestion.

Comment: *Suggestion to change Table 1 into a real pseudocode.*

Response: Following the reviewer's suggestion, we replaced Table 1 with a pseudocode-style representation presented as Figure 1. This change improves readability and more clearly conveys the step-by-step procedure for computing LAVI. This modification also ensures compatibility with the journal template, which does not support a dedicated algorithm environment.

Algorithm 1 Computation of LAVI for a seed node i

```
Input: A connected undirected graph G = (V, E), seed node i ∈ V
Parameter: Ordering mode ∈ {random, worst}
Output: LAVI value L(i)
Compute shortest-path distances d_i(v) from i to all v ∈ V using BFS
Group nodes by distance: π(i) = [S_0, S_1, ..., S_{d_max}]
for each layer S_ℓ do
    if mode = random then
        Randomly permute nodes in S_ℓ
    else if mode = worst then
        Sort nodes in S_ℓ in descending order of deg_G(v)
    end if
end for
Flatten π(i) into sequence (v_1, v_2, ..., v_|V|)
C_0 ← 0
for k = 1 to |V| do
    C_k ← C_{k-1} + deg_G(v_k)
end for
L(i) ← (1/|V|) Σ_{k=1}^{|V|} C_k
return L(i)
```

Fig 1. Pseudo-code for computing the LAVI value for a seed node *i*. Nodes are grouped by BFS distance from *i*, ordered within each layer, and evaluated via cumulative degree aggregation

Reviewer #2
We thank the reviewer for the positive evaluation and encouraging comments.

Reviewer #3
We thank the reviewer for evaluating our manuscript.
