# Supplementary material for: Impact of seed node position on network robustness under localized attacks
Source: PLoS One. 2026 Sep 22;21(9):e0358769. doi: 10.1371/journal.pone.0358769 (PMC13596812; doi:10.1371/journal.pone.0358769)
Supplement: Supplementary file 3 [file pone.0358769.s004.pdf]

Response to the Editor and Reviewers

We sincerely thank the Editor and the Reviewers for their careful evaluation of our manuscript and for their constructive comments. We have revised the manuscript extensively in response to these suggestions. In particular, we clarified the positioning and definition of the Localized Attack Vulnerability Index (LAVI), expanded the synthetic experiments to multiple network topologies, introduced a top-10% critical-seed evaluation for both synthetic and real-world networks, clarified the topology-dependent limitations of LAVI, and improved the description of the localized attack process and reproducibility. Our point-by-point responses are provided below.

Response to the Editor

Editor. The evaluation is not solid.

Answer. Thank you for this important comment. We agree that the evaluation in the previous version was insufficient to establish the generality and practical utility of LAVI. We have therefore substantially strengthened the evaluation in the revised manuscript.

First, we expanded the synthetic experiments beyond BA networks to include Erdős–Rényi (ER), Watts–Strogatz (WS), stochastic block model (SBM), and random geometric graph (RGG) networks, thereby covering homogeneous random, small-world, modular, and spatially embedded network structures. These experiments allow us to examine the extent to which the performance of LAVI depends on network topology.

Second, in addition to Pearson and Spearman correlations, we introduced a top-10% critical-seed evaluation for both synthetic and real-world networks. In this analysis, nodes are ranked according to each vulnerability metric or centrality measure, and the mean robustness R resulting from localized attacks initiated from the top-ranked nodes is compared. This directly evaluates whether each metric can identify particularly damaging seed nodes.

The additional experiments show that the LAVI-based measures generally identify more damaging seeds than conventional degree, closeness, and betweenness centralities in BA, ER, WS, and SBM networks, whereas closeness centrality performs better in the spatially constrained RGG network. In the real-world networks, a LAVI-based measure yields the lowest or tied-lowest mean robustness in six of the seven networks examined.

These results provide a broader and more direct evaluation of LAVI while also revealing that its effectiveness is topology dependent. We have revised the manuscript accordingly and moderated our claims to reflect these limitations.

These additions are described in the revised colored manuscript on Pages 7–8, Lines 243–285, for the expanded synthetic-network experiments and top 10% critical-seed evaluation, and on Pages 10–11, Lines 346–360, for the corresponding evaluation on real-world networks.

Response to Reviewer #3

Reviewer #3. Congrats, I think the manuscript can be accepted for publication in the present format. All the comments have been addressed.

Answer. Thank you very much for your positive evaluation of our manuscript and for confirming that your previous comments have been adequately addressed. We appreciate your careful review and constructive feedback throughout the review process.

Response to Reviewer #4

Reviewer #4, Comment 1. The reviewer believes that the novelty of this work is overstated. LAVI is essentially a distance-layered cumulative degree metric, so the claim that it is the first seed-level vulnerability index for localized attacks is not well-supported against existing localized attack literature. Also, the conceptual difference from prior propagation-aware node metrics remains unclear.

Answer. Thank you for this important comment. We agree that the novelty of LAVI should not be attributed simply to the use of distance layers or cumulative degree information. We have therefore revised the Introduction to moderate the novelty claim and clarify the positioning of our work. In particular, we no longer rely on an unsupported priority claim that LAVI is the first seed-level vulnerability index for localized attacks.

We now distinguish the role of a seed in localized attacks from that in conventional targeted attacks, network dismantling, and spreading processes such as epidemic or information diffusion. In a localized attack, the seed specifies the origin of a proximity-constrained removal process, and the damaged region expands outward according to the surrounding shell structure. Thus, the resulting damage depends not only on the structural importance of the seed itself but also on the connectivity encountered as the attack expands around that seed. This differs conceptually from propagation-aware metrics designed to identify influential spreaders in endogenous diffusion processes.

We also clarify that classical analytical studies of localized attacks have primarily characterized network-level robustness using randomly selected attack origins. Our contribution is therefore positioned more precisely as the explicit formulation and empirical evaluation of seed-specific vulnerability under localized attack dynamics, with LAVI serving as a simple structural proxy tailored to the shell-wise removal process.

Accordingly, we substantially revised the related discussion and the statement of our contributions in the Introduction (Pages 2–3, Lines 45–97 in revised colored manuscript).

Reviewer #4, Comment 2. The core assumption that cumulative half-link loss is equal to robustness loss no longer holds in networks dominated by modular/bridging structures. However, this metric has not been adjusted accordingly to take into account the structural bridges or community structures. This attack model only uses topological distance and ignores the spatial limitations of the actual local faults in the real world.

Answer. Thank you for this important comment. We agree that cumulative half-link loss should not be interpreted as being equivalent to actual robustness loss. LAVI is intended as a structural proxy for the severity of a localized attack, and its predictive performance depends on the extent to which cumulative connectivity loss reflects the resulting global fragmentation. We have revised the Discussion to clarify this interpretation and the associated limitations.

To examine the effect of meso-scale structure more explicitly, we extended the synthetic experiments to include a stochastic block model (SBM). In the SBM considered in this study, the LAVI-based measures remained more strongly associated with robustness than the conventional centrality measures. This indicates that the presence of community structure alone does not necessarily cause LAVI to perform poorly. However, LAVI does not explicitly distinguish the structural roles of individual links, such as inter-community bridges. The bio-diseasome network illustrates this limitation: seeds with similar cumulative connectivity-loss profiles can lead to substantially different fragmentation outcomes when the disruption of particular bridge-like connections strongly affects global connectivity. We therefore now explicitly state that LAVI should be interpreted as a proxy for structural vulnerability rather than as a direct measure of fragmentation and that its effectiveness may decrease in bridge-dependent network structures.

We also agree with the reviewer regarding spatial constraints. The localized attack model considered in this study is defined using shortest-path distance and therefore represents topological locality rather than geographical or physical locality. To examine topology dependence in spatially embedded networks, we added experiments using random geometric graphs (RGGs). In this topology, closeness

centrality outperformed the LAVI-based measures, suggesting that the global position of a seed can become particularly important when connectivity is strongly constrained by spatial embedding. Nevertheless, geographical distance is not explicitly incorporated into the attack process itself. We have therefore clarified this limitation in the Discussion and identified the extension of LAVI to localized attacks defined by geographical or other physical distance measures as an important direction for future work.

To address these points, we added the SBM and RGG experiments and their topology-dependent interpretation (Page 7, Lines 243–259; Page 8, Lines 270–285 in revised colored manuscript). We also expanded the Discussion to explicitly describe the limitations of LAVI in modular and bridge-dependent structures (Page 12, Lines 392–404) and to clarify that the present LA model represents topological rather than geographical locality (Page 12, Lines 424–433).

Reviewer #4, Comment 3. The evaluation in this work relies only on correlation coefficients without predictive performance metrics for identifying critical seeds. The dataset is small and limited, so it is not persuasive. However, controlled tests on modular, small-world, and large-scale spatial networks are missing.

Answer. Thank you for this important comment. We substantially expanded the evaluation in two directions.

First, we added controlled experiments on five representative synthetic network models: Barabási–Albert (BA), Erdős–Rényi (ER), Watts–Strogatz (WS), stochastic block model (SBM), and random geometric graph (RGG) networks. These models represent heterogeneous, homogeneous random, small-world, modular, and spatially embedded structures, respectively. We used comparable network sizes and mean degrees to facilitate comparisons across network topologies. The LAVI-based measures showed stronger Spearman correlations with robustness than the conventional centrality measures in the BA, ER, WS, and SBM networks, whereas closeness centrality performed better in the RGG network.

Second, following the reviewers' suggestions, we introduced a top-10% critical-seed evaluation. For each metric, nodes were ranked according to their predicted vulnerability, and localized attacks were initiated from the top 10% of nodes. We then compared the resulting mean robustness R, where smaller values indicate more damaging seed selections. In the BA, ER, WS, and SBM networks, the LAVI-based rankings produced lower mean robustness than the conventional degree, closeness, and betweenness centrality rankings. In the RGG network, closeness centrality performed best, again demonstrating the topology dependence of the relative performance.

We also applied the same critical-seed evaluation to the real-world networks. A LAVI-based measure yielded the lowest or tied-lowest mean robustness in six of the seven networks examined. These additional analyses complement the correlation-based evaluation by directly measuring whether each metric can identify particularly damaging seed nodes.

We agree that evaluation on an even broader collection of real-world networks would be valuable. In the present revision, we focused on expanding the controlled synthetic experiments so that the effects of several distinct structural properties could be evaluated systematically.

These additional evaluations are presented in the revised Results section: the controlled comparison across BA, ER, WS, SBM, and RGG networks appears on Pages 7–8, Lines 243–259, the top-10% critical-seed analysis for the synthetic networks on Pages 7–8, Lines 260–285, and the corresponding analysis for the real-world networks on Pages 10–11, Lines 346–360 in revised colored manuscript.

Reviewer #4, Comment 4. The manuscript does not provide quantitative topological thresholds to predict when LAVI is reliable. The declining performance in larger networks is observed but not theoretically explained.

Answer. Thank you for this important comment. We agree that the present study does not provide a quantitative topological threshold for determining when LAVI is reliable. We have revised the manuscript to state this limitation explicitly.

Existing analytical frameworks for localized attacks are primarily formulated for random networks with a prescribed degree distribution and characterize ensemble-level behavior by initiating the attack from a randomly selected root node [R1–R3]. This random-root assumption enables branching-process and generating-function treatments of the shell structure and the resulting giant connected component.

In contrast, the vulnerability considered in the present study is conditioned on a specific seed node. Such seed-specific behavior depends on the actual shell structure surrounding that node and, in general, on structural properties such as degree–degree correlations, bridge structure, and meso-scale organization that cannot be determined from the degree distribution alone. Consequently, deriving a quantitative reliability threshold for LAVI would require a theoretical framework that explicitly incorporates seed-conditioned shell structure and structural correlations. Such a framework is beyond the scope of the current analytical theory of localized attacks.

Furthermore, our additional experiments show that the performance of LAVI depends not only on network size but also on network topology. We have therefore revised the Discussion to avoid attributing the observed decline in performance solely to increasing network size. Instead, we now describe this observation as a topology-dependent limitation and identify the development of a seed-conditioned analytical framework incorporating degree correlations and meso-scale structure as an important direction for future work.

We revised the Discussion accordingly on Pages 11–12, Lines 382–423 in revised colored manuscript. In particular, Lines 382–390 discuss the relationship among network size, shell growth, and the dilution of seed-specific information; Lines 405–416 explicitly state the absence of a quantitative topological threshold and the need for a seed-conditioned analytical framework; and Lines 417–423 discuss the possible distance-truncated extension of LAVI.

References

[R1] Shao, J., Buldyrev, S. V., Braunstein, L. A., Havlin, S., and Stanley, H. E. "Structure of shells in complex networks." *Physical Review E*, 80, 036105 (2009).

[R2] Shao, S., Huang, X., Stanley, H. E., and Havlin, S. "Percolation of localized attack on complex networks." *New Journal of Physics*, 17, 023049 (2015).

[R3] Dong, G., et al. "Localized attack on networks with clustering." *New Journal of Physics*, 21, 013014 (2019).

Reviewer #4, Comment 5. The explanations of key symbols and the calculation steps of LAVI in the paper are not sufficient. The skip of the standardization process lacks statistical basis. Moreover, some implementation details are not described in sufficient detail, which is not enough to achieve complete reproducibility.

Answer. Thank you for this comment. We revised the Methods section to clarify the key symbols, shell construction, within-shell removal rules, and step-by-step calculation of LAVI. We also corrected the notation to clearly distinguish the seed-node index from the index of a node in the removal sequence.

We further clarified why LAVI is reported without normalization by $2M$, where M denotes the number of links. Dividing $\mathcal{L}(i)$ by $2M$ produces a normalized value in the range $[0, 1]$. However, because $2M$ is constant for all candidate seed nodes within a given network, this normalization is simply a positive linear rescaling. It therefore does not affect node rankings or the Pearson and Spearman correlations used in our analyses. It also does not affect the top-10% seed selection introduced in the

revised manuscript. Because the present study compares seed-node vulnerability within each network rather than absolute LAVI values across different networks, we report the unnormalized $\mathcal{L}(i)$.

Finally, to improve reproducibility, we expanded the implementation details in the manuscript and made the source code and accompanying data required to reproduce the LAVI calculations and localized attack simulations publicly available through Zenodo: `https://doi.org/10.5281/zenodo.21884803`.

The corresponding methodological revisions appear on Pages 3–5, Lines 104–187 in revised colored manuscript. In particular, the robustness definition and LA procedure are clarified on Pages 3–4, Lines 104–135; and the rationale for reporting the unnormalized LAVI on Page 5, Lines 173–181. The public availability of the data and source code is stated on Page 13, Lines 484–486.

Response to Reviewer #5

Reviewer #5, Comment 1. The paper relies exclusively on Pearson and Spearman correlations to demonstrate LAVI's superiority. While informative, correlations do not fully capture the practical utility of a node-level vulnerability index. A much stronger demonstration would be to rank all nodes by LAVI and by each centrality, then compare the average robustness R for the top-k (e.g., top 10%) seeds according to each metric. Such an analysis would directly show that LAVI-based prioritization identifies seeds that cause more severe damage, which is the ultimate goal of the index. I encourage the authors to include this evaluation.

Answer. Thank you for this valuable suggestion. Following the reviewer's recommendation, we added a top-10% critical-seed evaluation for both synthetic and real-world networks.

Specifically, all nodes were ranked according to each LAVI variant and conventional centrality measure, and localized attacks were initiated from the top 10% of nodes in each ranking. We then evaluated the mean robustness R of these attacks. Because a smaller R corresponds to greater structural damage, a ranking method that successfully identifies critical seeds is expected to produce a smaller mean R.

In the BA, ER, WS, and SBM networks, the LAVI-based rankings produced lower mean robustness than the conventional degree, closeness, and betweenness centrality rankings. In contrast, closeness centrality produced the lowest mean robustness in the RGG network. In the real-world networks, a LAVI-based measure yielded the lowest or tied-lowest mean robustness in six of seven cases.

Notably, the LAVI-based ranking also identified highly damaging seeds in the bio-diseasome network, even though its overall correlation with robustness was relatively weak. This result illustrates that global correlation and the ability to identify the most critical seeds capture complementary aspects of predictive performance. We have added this analysis and the corresponding discussion to the revised manuscript.

Following this suggestion, we added dedicated critical-seed identification analyses for both synthetic and real-world networks. The synthetic-network analysis is presented on Pages 7–8, Lines 260–285 (Table 3) in revised colored manuscript, and the real-world analysis on Pages 10–11, Lines 346–360 (Table 7).

Reviewer #5, Comment 2. The synthetic analysis is restricted to Barabási–Albert networks with a single parameter setting ($N = 100, 1000, 10000$, $m = 2$). This leaves open the question of whether LAVI's performance generalizes to other common network models (e.g., Erdős–Rényi, Watts–Strogatz, or spatial random graphs). Including at least one or two additional synthetic topologies with controlled experiments would significantly strengthen the generality claims.

Answer. Thank you for this suggestion. We expanded the synthetic analysis to five representative network models: BA, ER, WS, SBM, and RGG networks. To facilitate comparisons across network topologies, we used comparable network sizes and mean degrees, with $N = 1000$ and an average degree of approximately $\langle k \rangle = 6$.

We evaluated both Spearman correlations and top-10% critical-seed identification across these models. The LAVI-based measures performed better than the conventional centrality measures in both types of evaluation for the BA, ER, WS, and SBM networks. In contrast, closeness centrality performed best in the RGG network.

We now explicitly discuss this result as evidence that the effectiveness of LAVI is topology dependent. Thus, the expanded experiments show that the predictive ability of LAVI is not restricted to scale-free BA networks, while also identifying spatially constrained networks as an important case in which its relative advantage can decrease.

The expanded synthetic-network analysis is presented on Pages 7–8, Lines 243–285 in revised colored manuscript. The five network models and their controlled settings are introduced on Page 7, Lines 243–252, their correlation results are discussed on Lines 253–259, and the corresponding top-10% critical-seed results are presented on Pages 7–8, Lines 260–285.

Reviewer #5, Comment 3. Table 1 shows a noticeable drop in the Spearman correlation for LAVI as N increases from 100 to 10,000 (from -0.913 to -0.570). The authors attribute this to "local effects weakening" in larger networks. Does the "effective radius" of an attack (the number of shells l) become a more dominant factor than the seed position in very large networks? A brief discussion on whether LAVI should be calculated only up to a certain distance d for massive networks would be valuable.

Answer. Thank you for this insightful comment. We agree that the relationship between the effective attack radius and network size is a relevant issue. However, the present results are not sufficient to conclude that the attack radius becomes more important than the seed position as N increases. We have therefore revised the Discussion to avoid interpreting the observed decrease in correlation as a consequence of network size alone.

In networks with relatively short characteristic path lengths, successive distance shells can contain rapidly increasing numbers of nodes. Because the current LAVI integrates cumulative connectivity loss over the entire removal sequence, seed-specific structural information contained in the early shells may become progressively diluted when later shells contain substantial fractions of the network. The observed size dependence may therefore reflect the interplay among network size, characteristic path length, shell-growth behavior, and network topology rather than N alone.

Following the reviewer's suggestion, we also discuss a distance-truncated extension of LAVI, denoted by $\mathcal{L}^{(d)}(i)$, in which only shells within a maximum distance d from the seed are considered. Such a formulation could preserve information associated with the local neighborhood of the seed in very large networks. Determining an appropriate effective radius, for example based on the characteristic path length or the shell-growth profile, requires further theoretical and empirical investigation and is therefore left as an important direction for future work.

We revised the corresponding discussion on Pages 11–12, Lines 382–423 in revised colored manuscript. Lines 382–390 discuss the possible relationship between network size and shell-growth behavior, while Lines 417–423 introduce and discuss the distance-truncated extension $\mathcal{L}^{(d)}(i)$ suggested by the reviewer.

Response to Reviewer #6

Reviewer #6, Comment 1. The definition of the robustness index R in Eq. (1) appears to be mathematically ambiguous. Specifically, the variable q only denotes the number of removed nodes, but does not uniquely determine which nodes are removed during the localized attack process. Under the proposed LA model, the removed node set depends on the seed node, the shell expansion distance, and the within-shell removal order. Consequently, different combinations of q removed nodes may lead to substantially different largest connected components (LCCs), making $s_{\mathrm{LA}}(q)$ not uniquely defined.

In my opinion, the robustness metric R would become well-defined only if the removal process were explicitly parameterized by the shell distance d from the seed node, such that the removed node set uniquely corresponds to the union of all shells within distance d. Otherwise, the current formulation conflates attack progression by shell expansion with progression by removed-node count q, which introduces ambiguity into the definition of R. The authors are therefore encouraged to clarify whether R represents a realization-specific quantity, an expectation over multiple attack realizations, or an averaged metric over seed nodes and stochastic removal orders.

Answer. Thank you for pointing out this ambiguity. We agree that the original notation $s_{\mathrm{LA}}(q)$ was incomplete because the number of removed nodes q alone does not uniquely determine the removed node set. We have therefore revised Eq. (1) and the accompanying description to explicitly condition the robustness measure on both the seed node i and the node removal sequence π:

$$R(i,\pi) = \frac{1}{N}\sum_{q=1}^{N} s_{\mathrm{LA}}(q \mid i,\pi),$$

where $s_{\mathrm{LA}}(q \mid i,\pi)$ denotes the relative size of the largest connected component after the first q nodes in the removal sequence π have been removed.

For a given seed node i, the localized attack proceeds outward according to shortest-path distance from the seed. Nodes belonging to the same distance shell are removed in random order. Accordingly, once both the seed i and a particular removal sequence π are specified, the removed node set is uniquely determined at every removal step q. Thus, q represents the progression of a specified localized-attack realization, rather than uniquely defining the removed node set by itself.

We retain the node-removal count q as the progression variable rather than replacing it with the shell distance d. A single shell may contain multiple nodes, and the LCC can change as individual nodes within that shell are removed. Parameterizing the robustness only by complete shell removal would therefore omit these intermediate changes in connectivity. In addition, the conventional global robustness index is defined by accumulating the LCC size over successive node removals, and retaining q preserves consistency with this standard formulation.

We have also clarified how randomness in the within-shell removal order is handled. For a given seed and a particular sequence π, $R(i,\pi)$ is a realization-specific quantity. When multiple random within-shell removal sequences are generated for the same seed, the reported robustness is obtained by averaging $R(i,\pi)$ over these realizations. The robustness is therefore not averaged over different seed nodes; each seed is evaluated separately.

These revisions make the dependence of the robustness measure on the seed and removal realization explicit and remove the ambiguity in the original definition.

We revised Eq. (1) and the accompanying description in the Localized Attack and Robustness Index subsection (Pages 3–4, Lines 104–123 in revised colored manuscript). Specifically, the dependence of R and s_{LA} on the seed node i and removal sequence π is made explicit on Page 3, Lines 104–111, while the shell-wise removal procedure, the uniqueness of the removed node set for given (i,π), and the averaging over random within-shell realizations are clarified on Pages 3–4, Lines 112–123.
