# Supplementary material for: Impact of seed node position on network robustness under localized attacks
Source: PLoS One. 2026 Sep 22;21(9):e0358769. doi: 10.1371/journal.pone.0358769 (PMC13596812; doi:10.1371/journal.pone.0358769)
Supplement: Peer review history

# Peer review: Decision Letter 0

**DOI:** 10.1371/journal.pone.0358769.r001

**Authors:** Maher Maalouf

19 Dec 2025

Dear Dr. Chujyo,

Thank you for submitting your manuscript to PLOS ONE. After careful consideration, we feel that it has merit but does not fully meet PLOS ONE’s publication criteria as it currently stands. Therefore, we invite you to submit a revised version of the manuscript that addresses the points raised during the review process.

Please submit your revised manuscript by Feb 02 2026 11:59PM. If you will need more time than this to complete your revisions, please reply to this message or contact the journal office at plosone@plos.org. When you're ready to submit your revision, log on to https://www.editorialmanager.com/pone/ and select the 'Submissions Needing Revision' folder to locate your manuscript file.

- A letter that responds to each point raised by the academic editor and reviewer(s). You should upload this letter as a separate file labeled 'Response to Reviewers'.
- A marked-up copy of your manuscript that highlights changes made to the original version. You should upload this as a separate file labeled 'Revised Manuscript with Track Changes'.
- An unmarked version of your revised paper without tracked changes. You should upload this as a separate file labeled 'Manuscript'.

If you would like to make changes to your financial disclosure, please include your updated statement in your cover letter. Guidelines for resubmitting your figure files are available below the reviewer comments at the end of this letter.

If applicable, we recommend that you deposit your laboratory protocols in protocols.io to enhance the reproducibility of your results. Protocols.io assigns your protocol its own identifier (DOI) so that it can be cited independently in the future. For instructions see: https://journals.plos.org/plosone/s/submission-guidelines#loc-laboratory-protocols. Additionally, PLOS ONE offers an option for publishing peer-reviewed Lab Protocol articles, which describe protocols hosted on protocols.io. Read more information on sharing protocols at https://plos.org/protocols?utm_medium=editorial-email&utm_source=authorletters&utm_campaign=protocols.

We look forward to receiving your revised manuscript.

Kind regards,

Maher Maalouf, Ph.D.

Academic Editor

PLOS One

Journal Requirements:

When submitting your revision, we need you to address these additional requirements.

1. Please ensure that your manuscript meets PLOS ONE's style requirements, including those for file naming. The PLOS ONE style templates can be found at

https://journals.plos.org/plosone/s/file?id=wjVg/PLOSOne_formatting_sample_main_body.pdf and

https://journals.plos.org/plosone/s/file?id=ba62/PLOSOne_formatting_sample_title_authors_affiliations.pdf.

2. Please note that PLOS One has specific guidelines on code sharing for submissions in which author-generated code underpins the findings in the manuscript. In these cases, we expect all author-generated code to be made available without restrictions upon publication of the work. Please review our guidelines at https://journals.plos.org/plosone/s/materials-and-software-sharing#loc-sharing-code and ensure that your code is shared in a way that follows best practice and facilitates reproducibility and reuse.

3. Thank you for stating in your Funding Statement:

“Partial financial support was received from TechnoPro Holdings, Inc. FT and MC received this support. The funder had no role in study design, data collection and analysis, decision to publish, or preparation of the manuscript.’

Please provide an amended statement that declares *all* the funding or sources of support (whether external or internal to your organization) received during this study, as detailed online in our guide for authors at http://journals.plos.org/plosone/s/submit-now.  Please also include the statement “There was no additional external funding received for this study.” in your updated Funding Statement.

Please include your amended Funding Statement within your cover letter. We will change the online submission form on your behalf.

4. We note that your Data Availability Statement is currently as follows: [All relevant data are within the manuscript and its Supporting Information files.]

Please confirm at this time whether or not your submission contains all raw data required to replicate the results of your study. Authors must share the “minimal data set” for their submission. PLOS defines the minimal data set to consist of the data required to replicate all study findings reported in the article, as well as related metadata and methods (https://journals.plos.org/plosone/s/data-availability#loc-minimal-data-set-definition).

For example, authors should submit the following data:

- The values behind the means, standard deviations and other measures reported;

- The values used to build graphs;

- The points extracted from images for analysis.

Authors do not need to submit their entire data set if only a portion of the data was used in the reported study.

If your submission does not contain these data, please either upload them as Supporting Information files or deposit them to a stable, public repository and provide us with the relevant URLs, DOIs, or accession numbers. For a list of recommended repositories, please see https://journals.plos.org/plosone/s/recommended-repositories.

If there are ethical or legal restrictions on sharing a de-identified data set, please explain them in detail (e.g., data contain potentially sensitive information, data are owned by a third-party organization, etc.) and who has imposed them (e.g., an ethics committee). Please also provide contact information for a data access committee, ethics committee, or other institutional body to which data requests may be sent. If data are owned by a third party, please indicate how others may request data access.

5. Thank you for stating the following in the Acknowledgments Section of your manuscript:

‘Partial financial support was received from TechnoPro Holdings, Inc.”

We note that you have provided additional information within the Acknowledgements Section that is not currently declared in your Funding Statement. Please note that funding information should not appear in the Acknowledgments section or other areas of your manuscript. We will only publish funding information present in the Funding Statement section of the online submission form.

Please remove any funding-related text from the manuscript and let us know how you would like to update your Funding Statement. Currently, your Funding Statement reads as follows:

“Partial financial support was received from TechnoPro Holdings, Inc. FT and MC received this support. The funder had no role in study design, data collection and analysis, decision to publish, or preparation of the manuscript.”

Please include your amended statements within your cover letter; we will change the online submission form on your behalf.

6. If the reviewer comments include a recommendation to cite specific previously published works, please review and evaluate these publications to determine whether they are relevant and should be cited. There is no requirement to cite these works unless the editor has indicated otherwise.

[Note: HTML markup is below. Please do not edit.]

Reviewers' comments:

Reviewer's Responses to Questions

**Comments to the Author**

1. Is the manuscript technically sound, and do the data support the conclusions?

Reviewer #1: Yes

Reviewer #2: Yes

**********

2. Has the statistical analysis been performed appropriately and rigorously?

Reviewer #1: No

Reviewer #2: Yes

**********

3. Have the authors made all data underlying the findings in their manuscript fully available?

The PLOS Data policy

Reviewer #1: Yes

Reviewer #2: Yes

**********

4. Is the manuscript presented in an intelligible fashion and written in standard English?

Reviewer #1: Yes

Reviewer #2: Yes

**********

Reviewer #1: The manuscript introduces the Localized Attack Vulnerability Index (LAVI), a node-level metric designed to quantify the potential impact of localized attacks originating from specific seed nodes in complex networks. The authors show that LAVI correlates more strongly with robustness degradation under localized attacks than traditional centrality measures such as degree, closeness, and betweenness. The study includes experiments on both Barabási–Albert synthetic networks and a diverse set of real-world networks, demonstrating that LAVI consistently outperforms classical metrics in predicting vulnerability. While the idea is interesting and potentially valuable, the paper appears concise and lacks methodological depth and detailed discussion in several parts, limiting reproducibility and interpretability.

These are my suggestions to improve the paper:

1) Include a clearer comparison with related work. Situate LAVI more explicitly within the existing literature on network vulnerability and robustness, explaining what gaps it fills compared to previously proposed indices.

2) Expand the methodological details. The description of the LAVI metric and the localized attack model is concise. Adding step-by-step explanations or a small numerical example would significantly improve clarity and reproducibility. To improve clarity and reproducibility, I strongly recommend adding pseudocode for the main procedures.

3) Clarify the motivation for the two removal strategies. The rationale behind the “random-case” and “worst-case” removal orders could be better justified, including a clearer explanation of the real-world scenarios each scheme is intended to represent.

4) Strengthen the discussion of centrality metrics. The manuscript could benefit from a more thorough explanation of why classical centrality measures fail to correlate with robustness under localized attacks, possibly with illustrative examples or theoretical arguments.

5) Discuss exceptional cases more deeply. For networks in which LAVI shows weak correlations (e.g., the bio-diseasome network), expand the analysis to explore structural properties. I also would be curious about the results on a criminal network. Please see https://www.doi.org/10.1109/TIFS.2023.3256706.

6) Broaden the discussion section. The discussion is relatively short. Expanding it to cover practical implications, potential applications, and concrete examples of how LAVI could support infrastructure planning or risk assessment would strengthen the impact of the study. Explicitly addressing limitations would improve transparency and balance.

Reviewer #2: This manuscript titled "Impact of seed node position on network robustness under localized attacks" proposes the Localized Attack Vulnerability Index (LAVI), a novel node-level metric designed to assess the vulnerability of complex networks to spatially localized attacks (LAs). The authors conduct comprehensive numerical experiments on synthetic and real-world networks, demonstrating that LAVI correlates more strongly with robustness degradation than traditional centrality measures. While the study introduces a promising and timely metric for understanding network vulnerability under localized disruptions, some aspects could be clarified and strengthened to enhance the manuscript's contribution and impact.

1.The manuscript should include a more explicit justification for the choice of the link removal count as the core of LAVI, linking it more directly to practical implications for network functionality and resilience (e.g., service disruption, flow capacity) beyond just structural connectivity.

2.The interpretation of results for the bio-diseasome network is somewhat ambiguous, as LAVI performs weakly there. A deeper discussion on the potential reasons (e.g., community structure, degree distribution) and the implications for LAVI's generalizability across diverse network types would be valuable.

3.While the computational complexity is briefly noted, a discussion on the practical scalability and potential challenges of applying LAVI to extremely large-scale real-time systems (e.g., global internet) would strengthen the practical utility section.

4.The manuscript could be improved by adding a more detailed comparison or discussion on how LAVI specifically differs from or complements existing notions of "node influence" in failure propagation models beyond just centrality measures.

5.The invulnerability of networks is a hotspot in recent study, and some correlative discussions on the robustness of seeds may further improve the significance of this work, e.g., Swarm and Evolutionary Computation, vol. 87, p. 101542, 2024 and Knowledge-Based Systems, vol. 275, p. 110677, 2023.

**********

what does this mean?). If published, this will include your full peer review and any attached files.

If you choose “no”, your identity will remain anonymous but your review may still be made public.

**Do you want your identity to be public for this peer review?**  For information about this choice, including consent withdrawal, please see our Privacy Policy

Reviewer #1: No

Reviewer #2: No

**********

[NOTE: If reviewer comments were submitted as an attachment file, they will be attached to this email and accessible via the submission site. Please log into your account, locate the manuscript record, and check for the action link "View Attachments". If this link does not appear, there are no attachment files.]

To ensure your figures meet our technical requirements, please review our figure guidelines: https://journals.plos.org/plosone/s/figures

You may also use PLOS’s free figure tool, NAAS, to help you prepare publication quality figures: https://journals.plos.org/plosone/s/figures#loc-tools-for-figure-preparation.

NAAS will assess whether your figures meet our technical requirements by comparing each figure against our figure specifications.

# Author comment: Author response to Decision Letter 1

**DOI:** 10.1371/journal.pone.0358769.r002

4 Feb 2026

We would like to thank the Editor and the Reviewers for their careful reading of our manuscript and for their constructive and helpful comments. We have revised the manuscript accordingly and provided detailed responses to each comment below.

Reviewer #1:

1) Include a clearer comparison with related work. Situate LAVI more explicitly within the existing literature on network vulnerability and robustness, explaining what gaps it fills compared to previously proposed indices.

Ans. We revised the Introduction to include a clearer comparison with related work and to explicitly position LAVI within the existing literature on network robustness and vulnerability by clarifying the gap addressed by the proposed index. The corresponding changes were added in lines 29–65 on page 2.

2) Expand the methodological details. The description of the LAVI metric and the localized attack model is concise. Adding step-by-step explanations or a small numerical example would significantly improve clarity and reproducibility. To improve clarity and reproducibility, I strongly recommend adding pseudocode for the main procedures.

Ans. We added Table 1 to provide pseudocode and a step-by-step description of the main procedures, improving the clarity and reproducibility of the proposed method.

3) Clarify the motivation for the two removal strategies. The rationale behind the “random-case” and “worst-case” removal orders could be better justified, including a clearer explanation of the real-world scenarios each scheme is intended to represent.

Ans. We added a brief explanation clarifying the motivation of the random-case and worst-case removal strategies by relating them to stochastic damage scenarios and adversarial localized attacks, respectively (lines 123–128 on page 4).

4) Strengthen the discussion of centrality metrics. The manuscript could benefit from a more thorough explanation of why classical centrality measures fail to correlate with robustness under localized attacks, possibly with illustrative examples or theoretical arguments.

Ans. We expanded the discussion to explain that high-degree or high-betweenness nodes perform poorly under localized attacks, while high-closeness nodes show relatively better performance in some cases. We also discussed the behavior of LAVI in these examples and emphasized the fundamental difference between classical targeted attacks and localized attack propagation mechanisms. Specifically, the background motivation for degree- and betweenness-based targeted attacks is added in lines 29–34 on page 2, while the detailed discussion under localized attack scenarios is provided in lines 236–258 on page 8.

5) Discuss exceptional cases more deeply. For networks in which LAVI shows weak correlations (e.g., the bio-diseasome network), expand the analysis to explore structural properties. I also would be curious about the results on a criminal network. Please see https://www.doi.org/10.1109/TIFS.2023.3256706.

Ans. We expanded the analysis of the bio-diseasome network by adding network visualization and structural discussion to better explain the weak correlation observed for LAVI (lines 225–258 on pages 7–8). In addition, we discussed the potential application of LAVI to criminal networks as a future research direction in the Discussion section (lines 306–315 on pages 9–10).

6) Broaden the discussion section. The discussion is relatively short. Expanding it to cover practical implications, potential applications, and concrete examples of how LAVI could support infrastructure planning or risk assessment would strengthen the impact of the study. Explicitly addressing limitations would improve transparency and balance.

Ans. We expanded the Discussion section by adding practical implications and application examples of LAVI, as well as an explicit discussion of methodological limitations and computational considerations, in order to improve the balance and transparency of the manuscript (lines 288–315 on pages 9–10).

Reviewer #2:

1) The manuscript should include a more explicit justification for the choice of the link removal count as the core of LAVI, linking it more directly to practical implications for network functionality and resilience (e.g., service disruption, flow capacity) beyond just structural connectivity.

Ans. We added an explicit justification of the link removal count in the Method section by explaining its interpretation as a measure of cumulative connectivity loss during localized attack propagation (lines 134–143 on page 4). In addition, we clarified in the Discussion that LAVI is formulated from a structural connectivity perspective and discussed its relation to practical network functionality and potential extensions toward flow- and capacity-based models (lines 288–295 on page 9).

2) The interpretation of results for the bio-diseasome network is somewhat ambiguous, as LAVI performs weakly there. A deeper discussion on the potential reasons (e.g., community structure, degree distribution) and the implications for LAVI's generalizability across diverse network types would be valuable.

Ans. We expanded the discussion of the bio-diseasome network by adding a detailed structural analysis and visualization to explain the weak correlation observed for LAVI. We also discussed the implications of modular and bridge-dominated network structures for the generalizability of the proposed metric across different network types (lines 225–258 on pages 7–8).

3) While the computational complexity is briefly noted, a discussion on the practical scalability and potential challenges of applying LAVI to extremely large-scale real-time systems (e.g., global internet) would strengthen the practical utility section.

Ans. We expanded the Discussion section by adding a scalability-oriented discussion on the practical challenges of applying LAVI to large-scale and real-time network systems, including computational cost, dynamic topology changes, and data availability issues (lines 296–305 on page 9).

4) The manuscript could be improved by adding a more detailed comparison or discussion on how LAVI specifically differs from or complements existing notions of "node influence" in failure propagation models beyond just centrality measures.

Ans. We expanded the Introduction to clarify how LAVI differs from and complements existing node influence and failure propagation frameworks by emphasizing the fundamental distinction between global diffusion-based influence models and localized attack propagation scenarios (lines 29–65 on page 2).

5) The invulnerability of networks is a hotspot in recent study, and some correlative discussions on the robustness of seeds may further improve the significance of this work, e.g., Swarm and Evolutionary Computation, vol. 87, p. 101542, 2024 and Knowledge-Based Systems, vol. 275, p. 110677, 2023.

Ans. We added recent studies on robust and influential seed identification to the Introduction and discussed their relation to the present work in the context of network robustness and vulnerability analysis (lines 29–44 on page 2).

Attachment Submitted filename: plosone_letter.pdf

# Peer review: Decision Letter 1

**DOI:** 10.1371/journal.pone.0358769.r003

**Authors:** Feier Chen

19 Feb 2026

Dear Dr. Chujyo,

Thank you for submitting your manuscript to PLOS ONE. After careful consideration, we feel that it has merit but does not fully meet PLOS ONE’s publication criteria as it currently stands. Therefore, we invite you to submit a revised version of the manuscript that addresses the points raised during the review process.

Please submit your revised manuscript by Apr 05 2026 11:59PM. If you will need more time than this to complete your revisions, please reply to this message or contact the journal office at plosone@plos.org. When you're ready to submit your revision, log on to https://www.editorialmanager.com/pone/ and select the 'Submissions Needing Revision' folder to locate your manuscript file.

- A letter that responds to each point raised by the academic editor and reviewer(s). You should upload this letter as a separate file labeled 'Response to Reviewers'.
- A marked-up copy of your manuscript that highlights changes made to the original version. You should upload this as a separate file labeled 'Revised Manuscript with Track Changes'.
- An unmarked version of your revised paper without tracked changes. You should upload this as a separate file labeled 'Manuscript'.

If you would like to make changes to your financial disclosure, please include your updated statement in your cover letter. Guidelines for resubmitting your figure files are available below the reviewer comments at the end of this letter.

If applicable, we recommend that you deposit your laboratory protocols in protocols.io to enhance the reproducibility of your results. Protocols.io assigns your protocol its own identifier (DOI) so that it can be cited independently in the future. For instructions see: https://journals.plos.org/plosone/s/submission-guidelines#loc-laboratory-protocols. Additionally, PLOS ONE offers an option for publishing peer-reviewed Lab Protocol articles, which describe protocols hosted on protocols.io. Read more information on sharing protocols at https://plos.org/protocols?utm_medium=editorial-email&utm_source=authorletters&utm_campaign=protocols.

We look forward to receiving your revised manuscript.

Kind regards,

Feier Chen, Ph.D

Academic Editor

PLOS One

Journal Requirements:

If the reviewer comments include a recommendation to cite specific previously published works, please review and evaluate these publications to determine whether they are relevant and should be cited. There is no requirement to cite these works unless the editor has indicated otherwise.

Additional Editor Comments:

Major revision is needed in the following aspects.

Further justification and discussion of how network topology may influence the behavior of the index. Clearer description of innovation compared to the references.

[Note: HTML markup is below. Please do not edit.]

Reviewers' comments:

Reviewer's Responses to Questions

**Comments to the Author**

Reviewer #1: All comments have been addressed

Reviewer #2: All comments have been addressed

Reviewer #3: (No Response)

**********

2. Is the manuscript technically sound, and do the data support the conclusions?

Reviewer #1: Yes

Reviewer #2: Yes

Reviewer #3: Yes

**********

3. Has the statistical analysis been performed appropriately and rigorously?

Reviewer #1: Yes

Reviewer #2: Yes

Reviewer #3: Yes

**********

4. Have the authors made all data underlying the findings in their manuscript fully available?

The PLOS Data policy

Reviewer #1: Yes

Reviewer #2: Yes

Reviewer #3: Yes

**********

5. Is the manuscript presented in an intelligible fashion and written in standard English?

Reviewer #1: Yes

Reviewer #2: Yes

Reviewer #3: Yes

**********

Reviewer #1: All comments have been addressed but I would like to suggest to change Table 1 into a real pseudocode for the final version of the manuscript.

Reviewer #2: Thanks the author for addressing previous comments. I have no further ones. The manuscript has been distinctly improved.

Reviewer #3: (No Response)

**********

what does this mean?). If published, this will include your full peer review and any attached files.

If you choose “no”, your identity will remain anonymous but your review may still be made public.

**Do you want your identity to be public for this peer review?**  For information about this choice, including consent withdrawal, please see our Privacy Policy

Reviewer #1: No

Reviewer #2: No

Reviewer #3: No

**********

[NOTE: If reviewer comments were submitted as an attachment file, they will be attached to this email and accessible via the submission site. Please log into your account, locate the manuscript record, and check for the action link "View Attachments". If this link does not appear, there are no attachment files.]

To ensure your figures meet our technical requirements, please review our figure guidelines: https://journals.plos.org/plosone/s/figures

You may also use PLOS’s free figure tool, NAAS, to help you prepare publication quality figures: https://journals.plos.org/plosone/s/figures#loc-tools-for-figure-preparation.

NAAS will assess whether your figures meet our technical requirements by comparing each figure against our figure specifications.

# Author comment: Author response to Decision Letter 2

**DOI:** 10.1371/journal.pone.0358769.r004

18 Mar 2026

We sincerely thank the editor and the reviewers for their careful evaluation of our manuscript and for their constructive comments. We have revised the manuscript accordingly and summarize the main changes below, followed by point-by-point responses.

In this revision, we have made the following major changes:

1. We improved the clarity of the manuscript by revising the Introduction and Discussion to better explain the novelty of the proposed method and the role of network topology.

2. We expanded the Discussion section to explicitly address how network topology influences the performance of LAVI, including its behavior in heterogeneous and modular networks.

3. We revised the presentation of the algorithm by converting Table 1 into a pseudocode-style figure (Figure 1), as the journal template does not support a dedicated algorithm environment, thereby improving readability and clarity of the computational procedure.

4. We made minor edits throughout the manuscript to improve clarity, consistency, and readability.

We hope that these revisions satisfactorily address all concerns.

Response to the Editor

Comment:

Major revision is needed in the following aspects: Further justification and discussion of how network topology may influence the behavior of the index. Clearer description of innovation compared to the references.

Response:

We thank the editor for highlighting these important points.

Comment 1:

Further justification and discussion of how network topology may influence the behavior of the index.

Response:

We added discussions in Lines 283–300 on Page 9. To clarify the role of network topology, we expanded the Discussion section to explicitly explain how the performance of LAVI depends on structural properties of the network. In particular, we added new paragraphs that discuss why LAVI performs well in heterogeneous networks such as scale-free networks, where early link removal strongly affects global connectivity, and why its predictive power is reduced in networks with strong modular or bridge-dependent structures, where fragmentation is governed by specific inter-community connectors. These additions make explicit that the effectiveness of LAVI is inherently topology dependent.

Comment 2:

Clearer description of innovation compared to the references.

Response:

We revised the Introduction (Lines 67–75, Pages 2–3) to more clearly describe the novelty of this study. We clarified that, unlike conventional centrality measures that rely on static structural importance, the proposed LAVI incorporates propagation dynamics originating from a single seed node and enables seed-level vulnerability assessment under localized attack dynamics. We also revised the surrounding text to better distinguish our approach from existing centrality-based and optimization-based methods. This revision clarifies the distinction from prior work and addresses the editor’s concern regarding the novelty of the study.

Response to the Reviewers

Reviewer #1

We thank the reviewer for the positive evaluation and helpful suggestion.

Comment: Suggestion to change Table 1 into a real pseudocode.

Response: Following the reviewer’s suggestion, we replaced Table 1 with a pseudocode-style representation presented as Figure 1. This change improves readability and more clearly conveys the step-by-step procedure for computing LAVI. This modification also ensures compatibility with the journal template, which does not support a dedicated algorithm environment.

Reviewer #2

We thank the reviewer for the positive evaluation and encouraging comments.

Reviewer #3

We thank the reviewer for evaluating our manuscript.

Attachment Submitted filename: plosone_letter_auresp_2.pdf

# Peer review: Decision Letter 2

**DOI:** 10.1371/journal.pone.0358769.r005

**Authors:** Feier Chen

14 Jun 2026

Dear Dr. Chujyo,

Thank you for submitting your manuscript to PLOS ONE. After careful consideration, we feel that it has merit but does not fully meet PLOS ONE’s publication criteria as it currently stands. Therefore, we invite you to submit a revised version of the manuscript that addresses the points raised during the review process.

The evaluation is not solid.

Please submit your revised manuscript by Jul 29 2026 11:59PM. If you will need more time than this to complete your revisions, please reply to this message or contact the journal office at plosone@plos.org. When you're ready to submit your revision, log on to https://www.editorialmanager.com/pone/ and select the 'Submissions Needing Revision' folder to locate your manuscript file.

- A letter that responds to each point raised by the academic editor and reviewer(s). You should upload this letter as a separate file labeled 'Response to Reviewers'.
- A marked-up copy of your manuscript that highlights changes made to the original version. You should upload this as a separate file labeled 'Revised Manuscript with Track Changes'.
- An unmarked version of your revised paper without tracked changes. You should upload this as a separate file labeled 'Manuscript'.

If you would like to make changes to your financial disclosure, please include your updated statement in your cover letter. Guidelines for resubmitting your figure files are available below the reviewer comments at the end of this letter.

If applicable, we recommend that you deposit your laboratory protocols in protocols.io to enhance the reproducibility of your results. Protocols.io assigns your protocol its own identifier (DOI) so that it can be cited independently in the future. For instructions see: https://journals.plos.org/plosone/s/submission-guidelines#loc-laboratory-protocols. Additionally, PLOS ONE offers an option for publishing peer-reviewed Lab Protocol articles, which describe protocols hosted on protocols.io. Read more information on sharing protocols at https://plos.org/protocols?utm_medium=editorial-email&utm_source=authorletters&utm_campaign=protocols.

As the corresponding author, your ORCID iD is verified in the submission system and will appear in the published article. PLOS supports the use of ORCID, and we encourage all coauthors to register for an ORCID iD and use it as well. Please encourage your coauthors to verify their ORCID iD within the submission system before final acceptance, as unverified ORCID iDs will not appear in the published article. *Only*  the individual author can complete the verification step; PLOS staff *cannot*  verify ORCID iDs on behalf of authors.

We look forward to receiving your revised manuscript.

Kind regards,

Feier Chen, Ph.D

Academic Editor

PLOS One

Journal Requirements:

If the reviewer comments include a recommendation to cite specific previously published works, please review and evaluate these publications to determine whether they are relevant and should be cited. There is no requirement to cite these works unless the editor has indicated otherwise.

[Note: HTML markup is below. Please do not edit.]

Reviewers' comments:

Reviewer's Responses to Questions

**Comments to the Author**

Reviewer #3: All comments have been addressed

Reviewer #4: (No Response)

Reviewer #5: (No Response)

Reviewer #6: All comments have been addressed

**********

2. Is the manuscript technically sound, and do the data support the conclusions?

Reviewer #3: Yes

Reviewer #4: Partly

Reviewer #5: Yes

Reviewer #6: Yes

**********

3. Has the statistical analysis been performed appropriately and rigorously?

Reviewer #3: Yes

Reviewer #4: No

Reviewer #5: Yes

Reviewer #6: Yes

**********

4. Have the authors made all data underlying the findings in their manuscript fully available?

The PLOS Data policy

Reviewer #3: Yes

Reviewer #4: No

Reviewer #5: Yes

Reviewer #6: Yes

**********

5. Is the manuscript presented in an intelligible fashion and written in standard English?

Reviewer #3: Yes

Reviewer #4: Yes

Reviewer #5: Yes

Reviewer #6: Yes

**********

Reviewer #3: Congrats, I think the manuscript can be accepted for publication in the present format. All the comments have been addressed.

Reviewer #4: This manuscript proposes the Localized Attack Vulnerability Index (LAVI) to quantify node vulnerability under localized attacks and validates it on synthetic and real-world networks. Overall, the topic is meaningful for infrastructure resilience and network robustness, however, some major amendaments are necessary to further improve the quality of this manuscript. The detailed comments are provided below.

1.The reviewer believes that the novelty of this work is overstated. LAVI is essentially a distance-layered cumulative degree metric, so the claim that it is the first seed-level vulnerability index for localized attacks is not well-supported against existing localized attack literature. Also, the conceptual difference from prior propagation-aware node metrics remains unclear.

2.The core assumption that cumulative half-link loss is equal to robustness loss no longer holds in networks dominated by modular/bridging structures. However, this metric has not been adjusted accordingly to take into account the structural bridges or community structures. This attack model only uses topological distance and ignores the spatial limitations of the actual local faults in the real world.

3.The evaluation in this work relies only on correlation coefficients without predictive performance metrics for identifying critical seeds. The dataset is small and limited, so it is not persuasive. However, controlled tests on modular, small-world, and large-scale spatial networks are missing.

4.The manuscript does not provide quantitative topological thresholds to predict when LAVI is reliable. The declining performance in larger networks is observed but not theoretically explained.

5.The explanations of key symbols and the calculation steps of LAVI in the paper are not sufficient. The skip of the standardization process lacks statistical basis. Moreover, some implementation details are not described in sufficient detail, which is not enough to achieve complete reproducibility.

Reviewer #5: 1. The paper relies exclusively on Pearson and Spearman correlations to demonstrate LAVI’s superiority. While informative, correlations do not fully capture the practical utility of a node-level vulnerability index. A much stronger demonstration would be to rank all nodes by LAVI and by each centrality, then compare the average robustness $R$ for the top-k (e.g., top 10%) seeds according to each metric. Such an analysis would directly show that LAVI-based prioritization identifies seeds that cause more severe damage, which is the ultimate goal of the index. I encourage the authors to include this evaluation.

2. The synthetic analysis is restricted to Barabási–Albert networks with a single parameter setting (N=100,1000,10000, m=2). This leaves open the question of whether LAVI's performance generalizes to other common network models (e.g., Erdős–Rényi, Watts–Strogatz, or spatial random graphs). Including at least one or two additional synthetic topologies with controlled experiments would significantly strengthen the generality claims.

3. Table 1 shows a noticeable drop in the Spearman correlation for LAVI as $N$ increases from 100 to 10,000 (from -0.913 to -0.570). The authors attribute this to "local effects weakening" in larger networks. Does the "effective radius" of an attack (the number of shells $l$) become a more dominant factor than the seed position in very large networks? A brief discussion on whether LAVI should be calculated only up to a certain distance $d$ for massive networks would be valuable.

Reviewer #6: The definition of the robustness index R in Eq. (1) appears to be mathematically ambiguous. Specifically, the variable q only denotes the number of removed nodes, but does not uniquely determine which nodes are removed during the localized attack process. Under the proposed LA model, the removed node set depends on the seed node, the shell expansion distance, and the within-shell removal order. Consequently, different combinations of q removed nodes may lead to substantially different largest connected components (LCCs), making sLA(q) not uniquely defined.

In my opinion, the robustness metric R would become well-defined only if the removal process were explicitly parameterized by the shell distance d from the seed node, such that the removed node set uniquely corresponds to the union of all shells within distance d. Otherwise, the current formulation conflates attack progression by shell expansion with progression by removed-node count q, which introduces ambiguity into the definition of R. The authors are therefore encouraged to clarify whether R represents a realization-specific quantity, an expectation over multiple attack realizations, or an averaged metric over seed nodes and stochastic removal orders.

**********

what does this mean?). If published, this will include your full peer review and any attached files.

If you choose “no”, your identity will remain anonymous but your review may still be made public.

**Do you want your identity to be public for this peer review?**  For information about this choice, including consent withdrawal, please see our Privacy Policy

Reviewer #3: **Yes:** Mehrdad Agha Mohammad Ali Kermani

Reviewer #4: No

Reviewer #5: **Yes:** Jihui Han

Reviewer #6: No

**********

[NOTE: If reviewer comments were submitted as an attachment file, they will be attached to this email and accessible via the submission site. Please log into your account, locate the manuscript record, and check for the action link "View Attachments". If this link does not appear, there are no attachment files.]

To ensure your figures meet our technical requirements, please review our figure guidelines: https://journals.plos.org/plosone/s/figures

You may also use PLOS’s free figure tool, NAAS, to help you prepare publication quality figures: https://journals.plos.org/plosone/s/figures#loc-tools-for-figure-preparation.

NAAS will assess whether your figures meet our technical requirements by comparing each figure against our figure specifications.

# Author comment: Author response to Decision Letter 3

**DOI:** 10.1371/journal.pone.0358769.r006

13 Aug 2026

Response to the Editor and Reviewers

We sincerely thank the Editor and the Reviewers for their careful evaluation of our manuscript and for their constructive comments. We have revised the manuscript extensively in response to these suggestions. In particular, we clarified the positioning and definition of the Localized Attack Vulnerability Index (LAVI), expanded the synthetic experiments to multiple network topologies, introduced a top-10\% critical-seed evaluation for both synthetic and real-world networks, clarified the topology-dependent limitations of LAVI, and improved the description of the localized attack process and reproducibility. Our point-by-point responses are provided below.

Response to the Editor

Editor.

The evaluation is not solid.

Answer.

Thank you for this important comment. We agree that the evaluation in the previous version was insufficient to establish the generality and practical utility of LAVI. We have therefore substantially strengthened the evaluation in the revised manuscript.

First, we expanded the synthetic experiments beyond BA networks to include Erd\H{o}s--R\'enyi (ER), Watts--Strogatz (WS), stochastic block model (SBM), and random geometric graph (RGG) networks, thereby covering homogeneous random, small-world, modular, and spatially embedded network structures. These experiments allow us to examine the extent to which the performance of LAVI depends on network topology.

Second, in addition to Pearson and Spearman correlations, we introduced a top-10\% critical-seed evaluation for both synthetic and real-world networks. In this analysis, nodes are ranked according to each vulnerability metric or centrality measure, and the mean robustness $R$ resulting from localized attacks initiated from the top-ranked nodes is compared. This directly evaluates whether each metric can identify particularly damaging seed nodes.

The additional experiments show that the LAVI-based measures generally identify more damaging seeds than conventional degree, closeness, and betweenness centralities in BA, ER, WS, and SBM networks, whereas closeness centrality performs better in the spatially constrained RGG network. In the real-world networks, a LAVI-based measure yields the lowest or tied-lowest mean robustness in six of the seven networks examined.

These results provide a broader and more direct evaluation of LAVI while also revealing that its effectiveness is topology dependent. We have revised the manuscript accordingly and moderated our claims to reflect

these limitations.

These additions are described in the revised colored manuscript on Pages 7--8, Lines 243--285, for the expanded synthetic-network experiments and top 10\% critical-seed evaluation, and on Pages 10--11, Lines 346--360, for the corresponding evaluation on real-world networks.

Response to Reviewer #3

Reviewer #3.

Congrats, I think the manuscript can be accepted for publication in the present format. All the comments have been addressed.

Answer.

Thank you very much for your positive evaluation of our manuscript and for confirming that your previous comments have been adequately addressed. We appreciate your careful review and constructive feedback throughout the review process.

Response to Reviewer #4

Reviewer #4, Comment 1.

The reviewer believes that the novelty of this work is overstated. LAVI is essentially a distance-layered cumulative degree metric, so the claim that it is the first seed-level vulnerability index for localized attacks is not well-supported against existing localized attack literature. Also, the conceptual difference from prior propagation-aware node metrics remains unclear.

Answer.

Thank you for this important comment. We agree that the novelty of LAVI should not be attributed simply to the use of distance layers or cumulative degree information. We have therefore revised the Introduction to moderate the novelty claim and clarify the positioning of our work. In particular, we no longer rely on an unsupported priority claim that LAVI is the first seed-level vulnerability index for localized attacks.

We now distinguish the role of a seed in localized attacks from that in conventional targeted attacks, network dismantling, and spreading processes such as epidemic or information diffusion. In a localized attack, the seed specifies the origin of a proximity-constrained removal process, and the damaged region expands outward according to the surrounding shell structure. Thus, the resulting damage depends not only on the structural importance of the seed itself but also on the connectivity encountered as the attack expands around that seed. This differs conceptually from propagation-aware metrics designed to identify influential spreaders in endogenous diffusion processes.

We also clarify that classical analytical studies of localized attacks have primarily characterized network-level robustness using randomly selected attack origins. Our contribution is therefore positioned more precisely as the explicit formulation and empirical evaluation of seed-specific vulnerability under localized attack dynamics, with LAVI serving as a simple structural proxy tailored to the shell-wise removal process.

Accordingly, we substantially revised the related discussion and the statement of our contributions in the Introduction (Pages 2--3, Lines 45--97 in revised colored manuscript).

Reviewer #4, Comment 2.

The core assumption that cumulative half-link loss is equal to robustness loss no longer holds in networks dominated by modular/bridging structures. However, this metric has not been adjusted accordingly to take into account the structural bridges or community structures. This attack model only uses topological distance and ignores the spatial limitations of the actual local faults in the real world.

Answer.

Thank you for this important comment. We agree that cumulative half-link loss should not be interpreted as being equivalent to actual robustness loss. LAVI is intended as a structural proxy for the severity of a localized attack, and its predictive performance depends on the extent to which cumulative connectivity loss reflects the resulting global fragmentation. We have revised the Discussion to clarify this interpretation and the associated limitations.

To examine the effect of meso-scale structure more explicitly, we extended the synthetic experiments to include a stochastic block model (SBM). In the SBM considered in this study, the LAVI-based measures remained more strongly associated with robustness than the conventional centrality measures. This indicates that the presence of community structure alone does not necessarily cause LAVI to perform poorly. However, LAVI does not explicitly distinguish the structural roles of individual links, such as inter-community bridges. The bio-diseasome network illustrates this limitation: seeds with similar cumulative connectivity-loss profiles can lead to substantially different fragmentation outcomes when the disruption of particular bridge-like connections strongly affects global connectivity. We therefore now explicitly state that LAVI should be interpreted as a proxy for structural vulnerability rather than as a direct measure of fragmentation and that its effectiveness may decrease in bridge-dependent network structures.

We also agree with the reviewer regarding spatial constraints. The localized attack model considered in this study is defined using shortest-path distance and therefore represents topological locality rather than geographical or physical locality. To examine topology dependence in spatially embedded networks, we added experiments using random geometric graphs (RGGs). In this topology, closeness centrality outperformed the LAVI-based measures, suggesting that the global position of a seed can become particularly important when connectivity is strongly constrained by spatial embedding. Nevertheless, geographical distance is not explicitly incorporated into the attack process itself. We have therefore clarified this limitation in the Discussion and identified the extension of LAVI to localized attacks defined by geographical or other physical distance measures as an important direction for future work.

To address these points, we added the SBM and RGG experiments and their topology-dependent interpretation (Page 7, Lines 243--259; Page 8, Lines 270--285 in revised colored manuscript). We also expanded the Discussion to explicitly describe the limitations of LAVI in modular and bridge-dependent structures (Page 12, Lines 392--404) and to clarify that the present LA model represents topological rather than geographical locality (Page 12, Lines 424--433).

Reviewer #4, Comment 3.

The evaluation in this work relies only on correlation coefficients without predictive performance metrics for identifying critical seeds. The dataset is small and limited, so it is not persuasive. However, controlled tests on modular, small-world, and large-scale spatial networks are missing.

Answer.

Thank you for this important comment. We substantially expanded the evaluation in two directions.

First, we added controlled experiments on five representative synthetic network models: Barab\'asi--Albert (BA), Erd\H{o}s--R\'enyi (ER), Watts--Strogatz (WS), stochastic block model (SBM), and random geometric graph (RGG) networks. These models represent heterogeneous, homogeneous random, small-world, modular, and spatially embedded structures, respectively. We used comparable network sizes and mean degrees to facilitate comparisons across network topologies. The LAVI-based measures showed stronger Spearman correlations with robustness than the conventional centrality measures in the BA, ER, WS, and SBM networks, whereas closeness centrality performed better in the RGG network.

Second, following the reviewers' suggestions, we introduced a top-10\% critical-seed evaluation. For each metric, nodes were ranked according to their predicted vulnerability, and localized attacks were initiated from the top 10\% of nodes. We then compared the resulting mean robustness $R$, where smaller values indicate more damaging seed selections. In the BA, ER, WS, and SBM networks, the LAVI-based rankings produced lower mean robustness than the conventional degree, closeness, and betweenness centrality rankings. In the RGG network, closeness centrality performed best, again demonstrating the topology dependence of the relative performance.

We also applied the same critical-seed evaluation to the real-world networks. A LAVI-based measure yielded the lowest or tied-lowest mean robustness in six of the seven networks examined. These additional analyses complement the correlation-based evaluation by directly measuring whether each metric can identify particularly damaging seed nodes.

We agree that evaluation on an even broader collection of real-world networks would be valuable. In the present revision, we focused on expanding the controlled synthetic experiments so that the effects of several distinct structural properties could be evaluated systematically.

These additional evaluations are presented in the revised Results section: the controlled comparison across BA, ER, WS, SBM, and RGG networks appears on Pages 7--8, Lines 243--259, the top-10\% critical-seed analysis for the synthetic networks on Pages 7--8, Lines 260--285, and the corresponding analysis for the real-world networks on Pages 10--11, Lines 346--360 in revised colored manuscript.

Reviewer \#4, Comment 4.

The manuscript does not provide quantitative topological thresholds to predict when LAVI is reliable. The declining performance in larger networks is observed but not theoretically explained.

Answer.

Thank you for this important comment. We agree that the present study does not provide a quantitative topological

threshold for determining when LAVI is reliable. We have revised the manuscript to state this limitation explicitly.

Existing analytical frameworks for localized attacks are primarily formulated for random networks with a prescribed degree distribution and characterize ensemble-level behavior by initiating the attack from a randomly selected root node~[R1--R3]. This random-root assumption enables branching-process and generating-function treatments of the shell structure and the resulting giant connected component.

In contrast, the vulnerability considered in the present study is conditioned on a specific seed node. Such seed-specific behavior depends on the actual shell structure surrounding that node and, in general, on structural properties such as degree--degree correlations, bridge structure, and meso-scale organization that cannot be determined from the degree distribution alone. Consequently, deriving a quantitative reliability threshold for LAVI would require a theoretical framework that explicitly incorporates seed-conditioned shell structure and structural correlations. Such a framework is beyond the scope of the current analytical theory of localized attacks.

Furthermore, our additional experiments show that the performance of LAVI depends not only on network size but also on network topology. We have therefore revised the Discussion to avoid attributing the observed decline in performance solely to increasing network size. Instead, we now describe this observation as a topology-dependent limitation and identify the development of a seed-conditioned analytical framework incorporating degree correlations and meso-scale structure as an important direction for future work.

We revised the Discussion accordingly on Pages 11--12, Lines 382--423 in revised colored manuscript. In particular, Lines 382--390 discuss the relationship among network size, shell growth, and the dilution of seed-specific information; Lines 405--416 explicitly state the absence of a quantitative topological threshold and the need for a seed-conditioned analytical framework; and Lines 417--423 discuss the possible distance-truncated extension of LAVI.

References:

[R1] Shao, J., Buldyrev, S. V., Braunstein, L. A., Havlin, S., and Stanley, H. E. “Structure of shells in complex networks.” Physical Review E, 80, 036105 (2009).

[R2] Shao, S., Huang, X., Stanley, H. E., and Havlin, S. “Percolation of localized attack on complex networks.” New Journal of Physics, 17, 023049 (2015).

[R3] Dong, G., et al. “Localized attack on networks with clustering.” New Journal of Physics, 21, 013014 (2019)

Reviewer #4, Comment 5.

The explanations of key symbols and the calculation steps of LAVI in the paper are not sufficient. The skip of the standardization process lacks statistical basis. Moreover, some implementation details are not described in sufficient detail, which is not enough to achieve complete reproducibility.

Answer.

Thank you for this comment. We revised the Methods section to clarify the key symbols, shell construction, within-shell removal rules, and step-by-step calculation of LAVI. We also corrected the notation to clearly distinguish the seed-node index from the index of a node in the removal sequence.

We further clarified why LAVI is reported without normalization by $2M$, where $M$ denotes the number of links. Dividing $\mathcal{L}(i)$ by $2M$ produces a normalized value in the range $[0,1]$. However, because $2M$ is constant for all candidate seed nodes within a given network, this normalization is simply a positive linear rescaling. It therefore does not affect node rankings or the Pearson and Spearman correlations used in our analyses. It also does not affect the top-10\% seed selection introduced in the revised manuscript. Because the present study compares seed-node vulnerability within each network rather than absolute LAVI values across different networks, we report the unnormalized $\mathcal{L}(i)$.

Finally, to improve reproducibility, we expanded the implementation details in the manuscript and made the source code and accompanying data required to reproduce the LAVI calculations and localized attack simulations publicly available through Zenodo: {https://doi.org/10.5281/zenodo.21884803}.

The corresponding methodological revisions appear on Pages 3--5, Lines 104--187 in revised colored manuscript. In particular, the robustness definition and LA procedure are clarified on Pages 3--4, Lines 104--135; and the rationale for reporting the unnormalized LAVI on Page 5, Lines 173--181. The public availability of the data and source code is stated on Page 13, Lines 484--486.

Response to Reviewer #5

Reviewer #5, Comment 1.

The paper relies exclusively on Pearson and Spearman correlations to demonstrate LAVI's superiority. While informative, correlations do not fully capture the practical utility of a node-level vulnerability index. A much stronger demonstration would be to rank all nodes by LAVI and by each centrality, then compare the average robustness $R$ for the top-$k$ (e.g., top 10\%) seeds according to each metric. Such an analysis would directly show that LAVI-based prioritization identifies seeds that cause more severe damage, which is the ultimate goal of the index. I encourage the authors to include this evaluation.

Answer.

Thank you for this valuable suggestion. Following the reviewer's recommendation, we added a top-10\% critical-seed evaluation for both synthetic and real-world networks.

Specifically, all nodes were ranked according to each LAVI variant and conventional centrality measure, and localized attacks were initiated from the top 10\% of nodes in each ranking. We then evaluated the mean robustness $R$ of these attacks. Because a smaller $R$ corresponds to greater structural damage, a ranking method that successfully identifies critical seeds is expected to produce a smaller mean $R$.

In the BA, ER, WS, and SBM networks, the LAVI-based rankings produced lower mean robustness than the conventional degree, closeness, and betweenness centrality rankings. In contrast, closeness centrality produced the lowest mean robustness in the RGG network. In the real-world networks, a LAVI-based measure yielded the lowest or tied-lowest mean robustness in six of seven cases.

Notably, the LAVI-based ranking also identified highly damaging seeds in the bio-diseasome network, even though its overall correlation with robustness was relatively weak. This result illustrates that global correlation and the ability to identify the most critical seeds capture complementary aspects of predictive performance. We have added this analysis and the corresponding discussion to the revised manuscript.

Following this suggestion, we added dedicated critical-seed identification analyses for both synthetic and real-world networks. The synthetic-network analysis is presented on Pages 7--8, Lines 260--285 (Table 3) in revised colored manuscript, and the real-world analysis on Pages 10--11, Lines 346--360 (Table 7).

Reviewer #5, Comment 2.

The synthetic analysis is restricted to Barab\'asi--Albert networks with a single parameter setting ($N=100,1000,10000$, $m=2$). This leaves open the question of whether LAVI's performance generalizes to other common network models (e.g., Erd\H{o}s--R\'enyi, Watts--Strogatz, or spatial random graphs). Including at least one or two additional synthetic topologies with controlled experiments would significantly strengthen the generality claims.

Answer.

Thank you for this suggestion. We expanded the synthetic analysis to five representative network models: BA, ER, WS, SBM, and RGG networks. To facilitate comparisons across network topologies, we used comparable network sizes and mean degrees, with $N=1000$ and an average degree of approximately $\langle k\rangle=6$.

We evaluated both Spearman correlations and top-10\% critical-seed identification across these models. The LAVI-based measures performed better than the conventional centrality measures in both types of evaluation for the BA, ER, WS, and SBM networks. In contrast, closeness centrality performed best in the RGG network.

We now explicitly discuss this result as evidence that the effectiveness of LAVI is topology dependent. Thus, the expanded experiments show that the predictive ability of LAVI is not restricted to scale-free BA networks, while also identifying spatially constrained networks as an important case in which its relative advantage can

decrease.

The expanded synthetic-network analysis is presented on Pages 7--8, Lines 243--285 in revised colored manuscript. The five network models and their controlled settings are introduced on Page 7, Lines 243--252, their correlation results are discussed on Lines 253--259, and the corresponding top-10\% critical-seed results are presented on Pages 7--8, Lines 260--285.

Reviewer #5, Comment 3.

Table 1 shows a noticeable drop in the Spearman correlation for LAVI as $N$ increases from 100 to 10,000 (from $-0.913$ to $-0.570$). The authors attribute this to ``local effects weakening'' in larger networks. Does the ``effective radius'' of an attack (the number of shells $l$) become a more dominant factor than the seed position in very large networks? A brief discussion on whether LAVI should be calculated only up to a certain distance $d$ for massive networks would be valuable.

Answer.

Thank you for this insightful comment. We agree that the relationship between the effective attack radius and network

size is a relevant issue. However, the present results are not sufficient to conclude that the attack radius becomes more important than the seed position as $N$ increases. We have therefore revised the Discussion to avoid interpreting the observed decrease in correlation as a consequence of network size alone.

In networks with relatively short characteristic path lengths, successive distance shells can contain rapidly increasing numbers of nodes. Because the current LAVI integrates cumulative connectivity loss over the entire removal sequence, seed-specific structural information contained in the early shells may become progressively diluted when later shells contain substantial fractions of the network. The observed size dependence may therefore reflect the interplay among network size, characteristic path length, shell-growth behavior, and network topology rather than $N$ alone.

Following the reviewer's suggestion, we also discuss a distance-truncated extension of LAVI, denoted by $\mathcal{L}^{(d)}(i)$, in which only shells within a maximum distance $d$ from the seed are considered. Such a formulation could preserve information associated with the local neighborhood of the seed in very large networks. Determining an appropriate effective radius, for example based on the characteristic path length or the shell-growth profile, requires further theoretical and empirical investigation and is therefore left as an important direction for future work.

We revised the corresponding discussion on Pages 11--12, Lines 382--423 in revised colored manuscript. Lines 382--390 discuss the possible relationship between network size and shell-growth behavior, while Lines 417--423 introduce and discuss the distance-truncated extension $\mathcal{L}^{(d)}(i)$ suggested by the reviewer.

Response to Reviewer #6

Reviewer #6, Comment 1.

The definition of the robustness index $R$ in Eq.~(1) appears to be mathematically ambiguous. Specifically, the variable $q$ only denotes the number of removed nodes, but does not uniquely determine which nodes are removed during the localized attack process. Under the proposed LA model, the removed node set depends on the seed node, the shell expansion distance, and the within-shell removal order. Consequently, different combinations of $q$ removed nodes may lead to substantially different largest connected components (LCCs), making $s_{\mathrm{LA}}(q)$ not uniquely defined.

In my opinion, the robustness metric $R$ would become well-defined only if the removal process were explicitly parameterized by the shell distance $d$ from the seed node, such that the removed node set uniquely corresponds to the union of all shells within distance $d$. Otherwise, the current formulation conflates attack progression by shell expansion with progression by removed-node count $q$, which introduces ambiguity into the definition of $R$. The authors are therefore encouraged to clarify whether $R$ represents a realization-specific quantity, an expectation over multiple attack realizations, or an averaged metric over seed nodes and stochastic removal orders.

Answer.

Thank you for pointing out this ambiguity. We agree that the original notation $s_{\mathrm{LA}}(q)$ was incomplete because the number of removed nodes $q$ alone does not uniquely determine the removed node set. We have therefore revised Eq.~(1) and the accompanying description to explicitly condition the robustness measure on both the seed node $i$ and the node removal sequence $\pi$:

\[

R(i,\pi)

=

\frac{1}{N}

\sum_{q=1}^{N}

s_{\mathrm{LA}}(q\mid i,\pi),

\]

where $s_{\mathrm{LA}}(q\mid i,\pi)$ denotes the relative size of the largest connected component after the first $q$ nodes in the removal sequence $\pi$ have been removed.

For a given seed node $i$, the localized attack proceeds outward according to shortest-path distance from the seed. Nodes belonging to the same distance shell are removed in random order. Accordingly, once both the seed $i$ and a particular removal sequence $\pi$ are specified, the removed node set is uniquely determined at every removal

step $q$. Thus, $q$ represents the progression of a specified localized-attack realization, rather than uniquely defining the removed node set by itself.

We retain the node-removal count $q$ as the progression variable rather than replacing it with the shell distance $d$. A single shell may contain multiple nodes, and the LCC can change as individual nodes within that shell are removed. Parameterizing the robustness only by complete shell removal would therefore omit these intermediate changes in connectivity. In addition, the conventional global robustness index is defined by accumulating the LCC size over successive node removals, and retaining $q$

preserves consistency with this standard formulation.

We have also clarified how randomness in the within-shell removal order is handled. For a given seed and a particular sequence $\pi$, $R(i,\pi)$ is a realization-specific quantity. When multiple random within-shell removal sequences are generated for the same seed, the reported robustness is obtained by averaging $R(i,\pi)$ over these realizations. The robustness is therefore not averaged over different seed nodes; each seed is evaluated separately.

These revisions make the dependence of the robustness measure on the seed and removal realization explicit and remove the ambiguity in the original definition.

We revised Eq.~(1) and the accompanying description in the Localized Attack and Robustness Index subsection (Pages 3--4, Lines 104--123 in revised colored manuscript). Specifically, the dependence of $R$ and $s_{\mathrm{LA}}$ on the seed node $i$ and removal sequence $\pi$ is made explicit on Page 3, Lines 104--111, while the shell-wise removal procedure, the uniqueness of the removed node set for given $(i,\pi)$, and the averaging over random within-shell realizations are clarified on Pages 3--4, Lines 112--123.

Attachment Submitted filename: response_letter_plosone.pdf

# Peer review: Decision Letter 3

**DOI:** 10.1371/journal.pone.0358769.r007

**Authors:** Feier Chen

7 Sep 2026

Impact of seed node position on network robustness under localized attacks

PONE-D-25-55628R3

Dear Dr. Chujyo,

We’re pleased to inform you that your manuscript has been judged scientifically suitable for publication and will be formally accepted for publication once it meets all outstanding technical requirements.

Within one week, you’ll receive an e-mail detailing the required amendments. When these have been addressed, you’ll receive a formal acceptance letter and your manuscript will be scheduled for publication.

An invoice will be generated when your article is formally accepted. Please note, if your institution has a publishing partnership with PLOS and your article meets the relevant criteria, all or part of your publication costs will be covered. Please make sure your user information is up-to-date by logging into Editorial Manager at Editorial Manager® and clicking the ‘Update My Information' link at the top of the page. For questions related to billing, please contact billing support.

If your institution or institutions have a press office, please notify them about your upcoming paper to help maximize its impact. If they’ll be preparing press materials, please inform our press team as soon as possible -- no later than 48 hours after receiving the formal acceptance. Your manuscript will remain under strict press embargo until 2 pm Eastern Time on the date of publication. For more information, please contact onepress@plos.org.

Kind regards,

Feier Chen, Ph.D

Academic Editor

PLOS One

Additional Editor Comments (optional):

Reviewers' comments:

Reviewer's Responses to Questions

**Comments to the Author**

Reviewer #4: All comments have been addressed

Reviewer #5: All comments have been addressed

**********

2. Is the manuscript technically sound, and do the data support the conclusions?

Reviewer #4: Yes

Reviewer #5: Yes

**********

3. Has the statistical analysis been performed appropriately and rigorously?

Reviewer #4: Yes

Reviewer #5: Yes

**********

4. Have the authors made all data underlying the findings in their manuscript fully available?

The PLOS Data policy

Reviewer #4: Yes

Reviewer #5: Yes

**********

5. Is the manuscript presented in an intelligible fashion and written in standard English?

Reviewer #4: Yes

Reviewer #5: Yes

**********

Reviewer #4: The authors addressed all the issues. The paper can be accepted for publication in its current version.

Reviewer #5: (No Response)

**********

what does this mean?). If published, this will include your full peer review and any attached files.

If you choose “no”, your identity will remain anonymous but your review may still be made public.

**Do you want your identity to be public for this peer review?**  For information about this choice, including consent withdrawal, please see our Privacy Policy

Reviewer #4: No

Reviewer #5: **Yes:** Jihui Han

**********

# Editor report: Acceptance letter

**DOI:** 10.1371/journal.pone.0358769.r008

**Authors:** Feier Chen

PONE-D-25-55628R3

PLOS One

Dear Dr. Chujyo,

I'm pleased to inform you that your manuscript has been deemed suitable for publication in PLOS One. Congratulations! Your manuscript is now being handed over to our production team.

At this stage, our production department will prepare your paper for publication. This includes ensuring the following:

* All references, tables, and figures are properly cited

* All relevant supporting information is included in the manuscript submission,

* There are no issues that prevent the paper from being properly typeset

You will receive further instructions from the production team, including instructions on how to review your proof when it is ready. Please keep in mind that we are working through a large volume of accepted articles, so please give us a few days to review your paper and let you know the next and final steps.

Lastly, if your institution or institutions have a press office, please let them know about your upcoming paper now to help maximize its impact. If they'll be preparing press materials, please inform our press team within the next 48 hours. Your manuscript will remain under strict press embargo until 2 pm Eastern Time on the date of publication. For more information, please contact onepress@plos.org.

You will receive an invoice from PLOS for your publication fee after your manuscript has reached the completed accept phase. If you receive an email requesting payment before acceptance or for any other service, this may be a phishing scheme. Learn how to identify phishing emails and protect your accounts at https://explore.plos.org/phishing.

If we can help with anything else, please email us at customercare@plos.org.

Thank you for submitting your work to PLOS One and supporting open access.

Kind regards,

PLOS One Editorial Office Staff

on behalf of

Dr. Feier Chen

Academic Editor

PLOS One
